# Upregulation of Intermediate-Conductance Ca^2+^-Activated K^+^ Channels (KCNN4) in Porcine Coronary Smooth Muscle Requires NADPH Oxidase 5 (NOX5)

**DOI:** 10.1371/journal.pone.0105337

**Published:** 2014-08-21

**Authors:** Hope K. A. Gole, Darla L. Tharp, Douglas K. Bowles

**Affiliations:** 1 Department of Biomedical Sciences, University of Missouri Columbia, Columbia, Missouri, United States of America; 2 Dalton Cardiovascular Research Center, University of Missouri Columbia, Columbia, Missouri, United States of America; 3 Medical Pharmacology and Physiology, University of Missouri Columbia, Columbia, Missouri, United States of America; Albany Medical College, United States of America

## Abstract

**Aims:**

NADPH oxidase (NOX) is the primary source of reactive oxygen species (ROS) in vascular smooth muscle cells (SMC) and is proposed to play a key role in redox signaling involved in the pathogenesis of cardiovascular disease. Growth factors and cytokines stimulate coronary SMC (CSMC) phenotypic modulation, proliferation, and migration during atherosclerotic plaque development and restenosis. We previously demonstrated that increased expression and activity of intermediate-conductance Ca^2+^-activated K^+^ channels (KCNN4) is necessary for CSMC phenotypic modulation and progression of stenotic lesions. Therefore, the purpose of this study was to determine whether NOX is required for KCNN4 upregulation induced by mitogenic growth factors.

**Methods and Results:**

Dihydroethidium micro-fluorography in porcine CSMCs demonstrated that basic fibroblast growth factor (bFGF) increased superoxide production, which was blocked by the NOX inhibitor apocynin (Apo). Apo also blocked bFGF-induced increases in KCNN4 mRNA levels in both right coronary artery sections and CSMCs. Similarly, immunohistochemistry and whole cell voltage clamp showed bFGF-induced increases in CSMC KCNN4 protein expression and channel activity were abolished by Apo. Treatment with Apo also inhibited bFGF-induced increases in activator protein-1 promoter activity, as measured by luciferase activity assay. qRT-PCR demonstrated porcine coronary smooth muscle expression of NOX1, NOX2, NOX4, and NOX5 isoforms. Knockdown of NOX5 alone prevented both bFGF-induced upregulation of KCNN4 mRNA and CSMC migration.

**Conclusions:**

Our findings provide novel evidence that NOX5-derived ROS increase functional expression of KCNN4 through activator protein-1, providing another potential link between NOX, CSMC phenotypic modulation, and atherosclerosis.

## Introduction

One of the central components of cardiovascular disease (CVD) is atherosclerosis, which is a slow degenerative process characterized by remodeling of the arterial wall and formation of atherosclerotic plaques [1], [2]. A key to plaque development during atherosclerosis is vascular smooth muscle cell (SMC) phenotypic modulation, proliferation, and migration into the neointimal region of the vessel [3], [4], . The ability of vascular SMCs to undergo phenotypic modulation in response to physiological and pathophysiological cues is unique [6], [7], [8], [9]. The transition from a differentiated to a de-differentiated state in response to vascular injury, is marked by a suppression of SMC differentiation genes and an increased autocrine/paracrine generation of basic fibroblast growth factor (bFGF), platelet derived growth factor-BB (PDGF-BB), transforming growth factor (TGF-β), and angiotensin II (AngII) [7], [8], [9], [10], [11], [12].

We have previously shown that PDGF-BB induced coronary SMC (CSMC) phenotypic modulation requires the functional upregulation of intermediate-conductance Ca^2+^-activated K^+^ channels (KCNN4) [7]. KCNN4 are voltage-independent channels composed of six membrane-spanning domains, modulated by intracellular Ca^2+^ to induce hyperpolarization [13]. Within the vasculature these channels regulate membrane potential and calcium signaling in addition to playing a role in vasorelaxation and neointimal formation associated with CVD [13], [14], [15], [16]. Studies have shown that KCNN4 upregulation is required for mitogen-induced suppression of SMC markers as well as vascular SMC migration and proliferation, and has been shown to occur during atherosclerosis and restenosis indicating these channels play a key role in coronary plaque formation [7], [15], [17], [18]. KCNN4 upregulation has previously been shown to occur via transcriptional activation of activator protein-1 (AP-1) [7], [18], [19] and reduction in repressor element-1 silencing transcription factor (REST) [18], [20], [21]. AP-1 is a transcription factor complex composed of c-jun and c-fos dimers involved in the regulation of cell proliferation, growth, and differentiation [22], [23], [24], [25]. Studies have shown that in addition to being activated by growth factors, serum, and cytokines [24], the AP-1 components are also increased following coronary angioplasty [18]. These results support the idea that AP-1 is a critical component of signaling pathways involved in KCNN4 regulation [13].

Along with multiple humoral factors, research has shown that reactive oxygen species (ROS) also play a role in vascular SMC phenotypic modulation and proliferation associated with the development of atherosclerosis and post-angioplasty restenosis [26], [27]. The primary source of ROS in vascular SMCs is the enzyme nicotinamide adenine dinucleotide phosphate (NADPH) oxidase, abbreviated NOX, which transfers electrons across biological membranes to oxygen forming superoxide (O_2_^.−^) [28], [29], [30], [31], [32]. NOX is a multimeric enzyme composed of plasma membrane associated-proteins as well as cytosolic factors [33], [34], [35], [36], that has been shown to be activated by numerous growth factors including vascular endothelial growth factor (VEGF), PDGF-BB, and endothelial growth factor (EGF) [26], [37], [38], [39]. NOX activation results in increased mRNA expression through transcriptional upregulation of redox-sensitive second messenger systems (e.g. MAP kinase activation), or transcription factor activation including nuclear factor-kappaB (NFkB), protein 53 (p53), and AP-1 [40], [41].

Of the seven NOX isoforms, human cardiovascular tissues express NOX1, NOX2, NOX4, and NOX5 [26], [42], [43]. Studies have shown that each isoform has varying expression levels, is differentially regulated, and thought to play a unique role in cardiovascular disease [43], [44], [45]. Research to date indicates that NOX1 is upregulated during vascular injury, atherosclerosis, and hypertension [43], [44], [46], [47], [48]; NOX2 is upregulated during atherosclerosis and vascular injury [44], [46], [49], [50]; NOX4 expression is upregulated during the re-differentiation phase of neointimal formation and the late stages of restenosis [9], [44], [46]; and NOX5 is upregulated during atherosclerosis [43], [51].

While NOX has been shown to act through multiple signaling pathways, the precise mechanisms by which NOX contributes to vascular SMC phenotypic modulation and proliferation remains unclear. Given the central role of KCNN4 in phenotypic modulation and neointimal formation [7], [18] and the recently described role of NOX5 in human SMC proliferation and atherosclerosis [51], [52], we tested the hypothesis that NOX5 is required for transcriptional upregulation of KCNN4 through AP-1. Compared to the other isoforms, NOX5 is of particular interest given it is uniquely regulated by calcium and remains relatively understudied as it is not expressed in rodents [44], [53]. Our results demonstrate that similar to humans, all four cardiovascular NOX isoforms, including NOX5, are expressed in porcine coronary smooth muscle (CSM) and NOX5-derived ROS are required for growth factor-induced KCNN4 upregulation, providing a novel link between NOX5, CSMC phenotypic modulation, and atherosclerosis.

## Materials and Methods

### Ethics Statement

Animal protocols were approved by the University of Missouri Animal Care and Use Committee in accordance with the United States National Institutes of Health “Guide for the Care and Use of Laboratory Animals”. Adequate depth of anesthesia was determined by pedal reflex.

### Isolation of coronary arteries

Yucatan swine obtained from the Sinclair Research Farm (Columbia, MO, USA) were anesthetized with telazol (5 mg/kg) and xylazine (2.25 mg/kg), followed by administration of heparin (1000 U/kg). Swine were euthanized by removal of the heart, which was immediately placed in 4°C physiological saline solution (PSS). The right coronary artery (RCA) was isolated, cleaned of fat and connective tissue, placed in low Ca^2+^ PSS containing 20 mM HEPES and stored at 4°C until use (0–1 days).

### Porcine CSMC culture

Primary CSMCs were isolated from the medial portion of a castrated male yucatan porcine RCA following removal of adventitia and mechanical denudation to remove endothelium, as previously described [7], [54]. Cells were initially plated at 1.5×10^4^ cells/cm^2^ in custom DMEM/F-12 media (GIBCO RR070050) without L-valine, containing pyridoxine HCl, 100 U/mL pen/strep, 1.6 mM L-glutamine, and 10% FBS for 4–6 days until post-confluent. Media was changed every two days. Subsequent passages were plated at the same density in DMEM/F-12 media (GIBCO 11320-033) containing 100 U/mL pen/strep, 1.6 mM L-glutamine, and 10% FBS. Cells (passage 2–6) were then serum starved 5 days to maximize smooth muscle differentiation marker gene [smooth muscle alpha-actin (SMαA), smooth muscle myosin heavy chain (SMMHC), and smoothelin (SE)] expression levels, as previously shown [7].

### ROS detection

O_2_^.−^ anion production was determined using dihydroethidium (DHE) micro-fluorography. In the presence of the O_2_^.−^ anion, DHE (which is freely permeable to cells) is oxidized to 2-hydroxyethidium [55]. The 2-hydroxyethidium becomes trapped intracellularly, producing fluorescence at an excitation wavelength of 488–520 nm with an emission spectrum of 610 nm [56], [57]. Unlike techniques utilizing lucigenin, DHE has little capacity for redox cycling thus the fluorescence detected correlates well to the level of cellular O_2_^.−^. Cultured CSMCs were grown to confluence on cover slips, serum starved for 5 days, and treated with bFGF (25 ng/mL, Upstate), bFGF+apocynin (Apo 1.0 mM, Sigma), bFGF+Tempol (1.0 mM, Sigma), Tempol, or Apo for 24 hours. Apo is used as an NOX inhibitor, disrupting the assembly of cytosolic and membrane components necessary for enzymatic activity [44], [47]. Under minimal light conditions, cells were incubated with DHE (10 µM, Sigma) for 30 minutes at 37°C, with the addition of the nuclear stain DAPI (10 mM, Molecular Probes) for the last 15 minutes. Following incubation, the cover slips were mounted on microscope slides for imaging. Images were obtained at the Molecular Cytology Core (University of Missouri, Columbia).

### Quantitative reverse-transcriptase PCR (qRT-PCR)

qRT-PCR was performed as previously described [7], [58], [59]. Cells in TRIzol were quick frozen in liquid nitrogen, stored at −80°C until processed, and total RNA was isolated according to the published TRIzol protocol. cDNA was transcribed from total RNA using Applied Bioscience high-capacity cDNA reverse transcription kit and qRT-PCR was performed on a MyiQ iCycler (Bio-Rad, model 170-9770). Each 25 µL reaction contained 1X Syber Green Master Mix (Bio-Rad), 0.8 µM forward and reverse primers, and 1 µg of cDNA. Each reaction was initiated by a 95°C hold for 3 minutes in order to activate heat stable Taq polymerase, and reaction conditions were optimized for each set of primers: KCNN4, NOX1, NOX2, NOX4, NOX5, and 18S (for primer sequences refer to Table 1). Target gene expression was normalized to 18S ribosomal RNA using the 2^−ΔΔCT^ method [60], [61]. To ensure reliable comparison between target genes, PCR linearity and efficiency was verified by creating a standard curve plotting the critical threshold versus log of the dilution of cDNA. Each primer set had a slope of 3.3±0.3, indicating a 90–100% efficiency, and an R^2^ value >0.99 providing strong confidence of correlating values.

**Table 1:** Primer Sequences (5′ to 3′).

Target Gene	Forward Primer	Reverse Primer
KCNN4	CCC ATC ACA TTC CTG ACC AT	GTC CTT CCT ACG CGT GTG TT
NOX1	AAT GGC ATC CCT TTA CCC TGA CCT	CTT GGA ACT GGC GAA TGC TGT TGT
NOX2	TAA GCA GTG CAT CTC CAA CTC CGA	GGC ATT ATC TGG GCA TTT GGC AGT
NOX4	TGC ATA ACA AGT TTT GGC AAG A	ATC CCA TCT GTT TGA CTG AG
NOX5	TGC TGA GAG ATT CTT CGC CCT CTT	AGG AAC TTG AGT TTG TCC GTG GGA
18S	CGG CTA CCA CAT CCA AGG AA	AGC TGG AAT TAC CGC GGC

### Immunohistochemistry

Immunohistochemistry was performed as previously described [59]. RCA sections were incubated with avidin–biotin two-step blocking solution (Vector SP-2001) to inhibit background staining and 3% hydrogen peroxide to inhibit endogenous peroxidase. Non-serum protein block (Dako X909) was then applied to inhibit non-specific protein binding. Sections were incubated at 4°C overnight with primary antibodies KCNN4 (1∶600, Chemicon), nitrotyrosine (1∶400, Chemicon), or SMαA (1∶200, DAKO). After appropriate washing steps, sections were incubated with biotinylated secondary antibody in phosphate-buffered saline containing 15 mM sodium azide and peroxidase-labeled streptavidin (Dako LSAB+ kit, peroxidase, K0690). Diaminobenzidine (DAB, Dako) was applied 5 minutes for visualization of the reaction product, sections were then counterstained with haematoxylin, dehydrated, and coverslipped. Images of the sections were obtained using an Olympus BX40 photomicroscope and Spot Insight Color camera (Diagnostic Instruments). The relative area and mean density of positive staining for KCNN4 were determined for each section of interest utilizing ImagePro Plus (Media Cybernetics).

### Whole cell voltage clamp

Whole cell K^+^ current (I_K_) was measured as previously described [7], [62], [63]. Cultured CSMCs were trypsinized then suspended in a low-Ca^2+^ PSS containing 20 mM HEPES and stored at 4°C until use (0–1 days). Normal PSS containing (in mM) 2 CaCl_2_, 10 glucose, 10 HEPES, 5 KCl, 1 MgCl_2_, and 138 NaCl, pH 7.4 was used to superfuse the cells at room temperature under gravity flow. Pipettes (2–6 MΩ) were filled with solution containing (in mM) 0.50 CaCl_2_ (0.5 µM free Ca^2+^), 100 KCl, 10 NaCl, 1 MgCl_2_, 10 HEPES, and 10 K2EGTA, with pH 7.1. To confirm calcium-sensitivity, potassium currents were also measured under nominally calcium free internal (Ca_i_) conditions using normal PSS containing (in mM) 0.1CaCl_2_, 10 glucose, 20 HEPES, 5 KCl, 1 MgCl_2_, and 138 NaCl, pH 7.4 as the superfusate, and (in mM) 120 KCl, 10 NaCl, 1 MgCl_2_, 10 HEPES, 10 K_2_EGTA, pH 7.1 as the pipette solution. After seal formation (seal resistance >1 GΩ), series resistance was monitored for determination of sufficient whole cell access (series resistance below 25 MΩ). From a holding potential of –80 mV, currents were elicited by 500-ms step depolarizations to potentials ranging from –70 mV to +100 mV (in 10 mV increments). Current was recorded in control cells, cells treated with bFGF (25 ng/mL), or cells treated with bFGF+Apo (1.0 mM) for 24 hours, both before and after addition of TRAM-34 (a selective KCNN4 channel blocker, 100 nM) to the superfusate. To account for differences in cell membrane surface area, current densities (pA/pF) were obtained for each cell by normalization of whole cell current to cell capacitance. An initial seal was obtained in 76 cells (26 control, 26 bFGF, 14 bFGF+Apo, and 10 bFGF-Ca^2+^_i_), and 51 cells (16 control, 17 bFGF, 10 bFGF+Apo, and 8 bFGF-Ca^2+^_i_) were selected for difference current calculations. Failure to maintain a tight seal throughout the entire protocol resulting in significant current leak across the cell membrane lead to a total of 25 cells being rejected (10 control, 9 bFGF, 4 bFGF+Apo, and 2 bFGF-Ca^2+^_i_). Data acquisition and analysis were accomplished using pCLAMP 8.0 software (Axon Instruments).

### AP-1 Plasmid Transfection

4 day serum-starved cultured CSMCs were trypsinized, placed in basic smooth muscle nucleofection solution (Amaxa), and transfected with pAP-1 Luc Cis-Reporter plasmid in which firefly luciferase gene expression in the reporter plasmid is controlled by a synthetic promoter containing direct repeats of the AP-1 transcription recognition sequence (1.0ug, Stratagene 219074). In addition to the desired plasmids, each sample contained 1×10^6^ cells, 100uL of nucleofection solution and 0.05uL of Renilla luciferase. Samples were placed in electroporation cuvettes (Amaxa) and nucleofected using program D-33 of the Amaxa nucleofector device, as previously described [7]. Following nucleofection, cells were immediately placed in RPMI media, incubated at 37°C and 5% CO_2_ for 15 minutes, then plated in 6-well plates containing DMEM/F-12 media (GIBCO 11320-033) with 100 U/mL pen/strep, 1.6 mM L-glutamine, and 10% FBS. Upon adherence (∼4 hours), cells were serum-starved for 4 hours then incubated with drug treatments for an additional 24 hours in serum free media. Following a 24-hour treatment period, the luciferase activity assay was performed.

### AP-1 Luciferase Activity Assay

After treatment, nucleofected CSMCs were washed in PBS and lysed with luciferase assay cell lysis buffer (Stratagene). The cell lysate was mixed with an equal amount of luciferase assay reagent (Stratagene) and luciferase activity measured using a luminometer (Monolight 2010; Analytical Luminescence Laboratory, SanDiego, CA). Results were expressed as a ratio of the firefly luciferase to Renilla luciferase luminescence normalized to control cells (fold induction).

### Adenovirus and shRNA

Replication-deficient adenovirus for GFP-tagged NOX5 shRNA (AdNOX5shRNA; Open Biosystems, GIPZ Human clone V3LHS_353966) was generated using AdEasy adenoviral vector systems (Agilent). CSMCs were serum-starved for 24 hours, and then infected with AdNox5shRNA at an MOI of 100 plaque forming units for 48 hours at 37°C. Cells were cultured in non-virus containing serum-free media for an additional 4 days, and then treated with bFGF (25 ng/mL) for 24 hours. Following treatment, cells were frozen in TRIzol and qRT-PCR was performed as described above.

### Nucleofection and siRNA application

Prior to performing nucleofection with NOX isoform siRNA’s, time course experiments were conducted to determine the duration of gene knockdown for each isoform. 90–100% confluent, 4 day serum-starved CSMCs were trypsinized and placed in basic smooth muscle cell nucleofection solution (Amaxa) with either a negative control siRNA, NOX2 siRNA, or NOX4 siRNA. Nucleofection was performed as described above. Each sample contained 1.0×10^6^ cells, 100 µL of nucleofection solution, and 2.5 ng of siRNA. Following treatment, cells were frozen in TRIzol and qRT-PCR was performed as described above. The NOX isoform siRNA’s were custom designed by Ambion (Table 2) and the negative control siRNA was purchased from Ambion (Silencer Negative control siRNA #1).

**Table 2:** NOX siRNA sequences (5′ to 3′).

Target Gene	Sense	Antisense
NOX2	GGA UGG AGG UGG GAC AAU Att	UAU UGU CCC ACC UCC AUC Ctg
NOX4	GUG UCC UAC UGA AAC CAA Att	UUU GGU UUC AGU AGG ACA Cat

### Chemotaxis

Seven days after transduction with control adenovirus or AdNOX5shRNA, postconfluent porcine coronary SMCs were plated at 30,000–40,000 cells/well in the upper chamber of a 10-µm-pore, 96-well chemotaxis chamber (Millipore). The following solutions were placed in the lower chamber (diluted in serum-free media): vehicle, PDGF-BB (30 ng/ml), and PDGF-BB+TRAM-34 (100 nM). The chamber was placed at 37°C for 4 h. Cells from the upper chamber were removed, and the filters were stained using Diff-Quik staining kit (Fisher Scientific). The migrated cells in a 20x field were manually counted.

### Statistics

All data are presented as mean±SE. One-way ANOVA was used for all group comparisons, and significance was defined as P<0.05.

## Results

### bFGF increased NOX-derived O_2_^.−^ production

**Figure 1:**
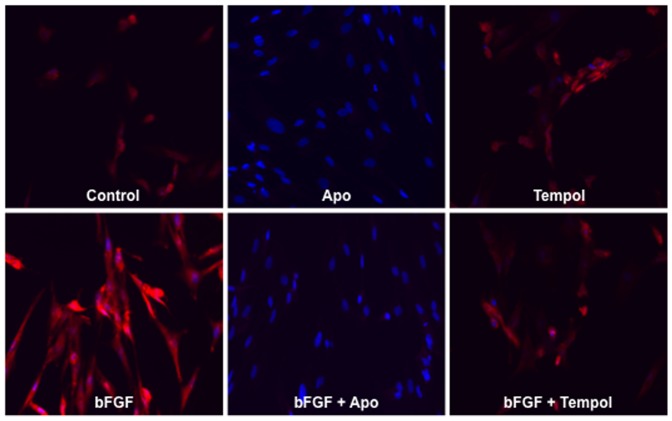
bFGF increased NOX-derived O_2_^.−^ **production.** bFGF (25 ng/mL) treatment for 24 hours increased DHE fluorescence (red) in CSMCs compared to control. Addition of the NOX inhibitor Apo (1.0 mM) and superoxide scavenger Tempol (1.0 mM) blocked bFGF induced O_2_^.−^ production. DAPI stained nuclei are shown in blue. Data presented are representative images of three different experiments.

To determine if bFGF upregulates NOX-derived O_2_^.−^ production, cultured CSMCs were treated with bFGF, the NOX inhibitor Apo, and the O_2_^.−^ scavenger Tempol. DHE micro-fluorography showed that bFGF increased O_2_^.−^ production, Apo inhibited both bFGF induced and basal O_2_^.−^ production, and Tempol inhibited bFGF induced O_2_^.−^ production (Figure 1).

### Inhibition of NOX prevented bFGF upregulation of KCNN4 mRNA expression

**Figure 2:**
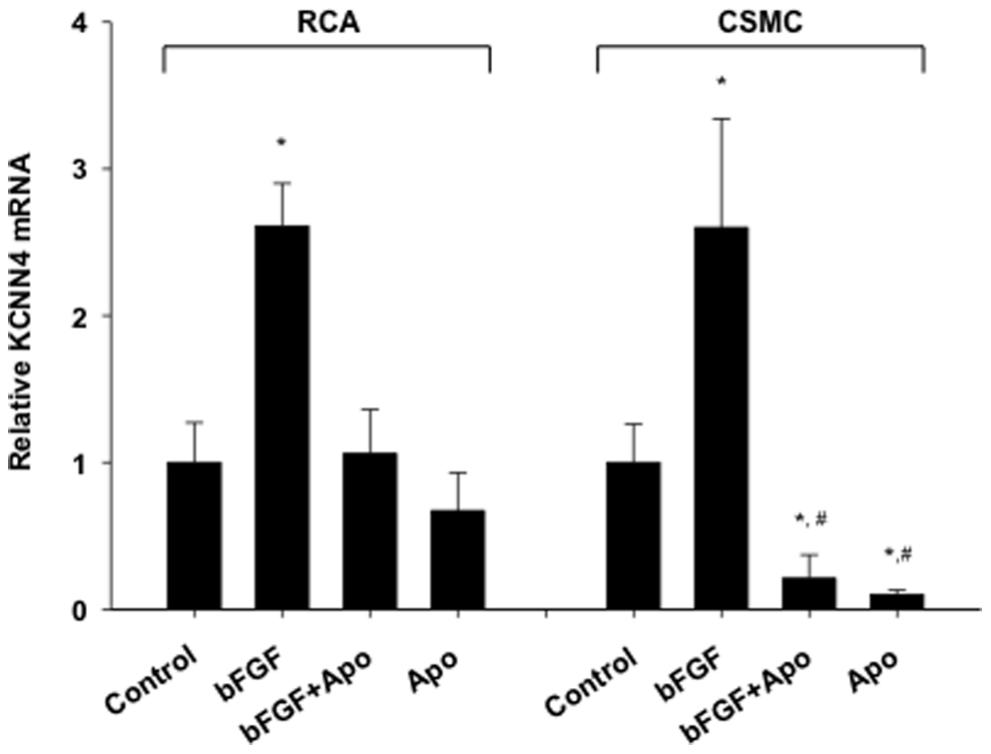
Inhibition of NOX prevented bFGF upregulation of KCNN4 mRNA expression. KCNN4 mRNA expression increased approximately 2.5 fold in both RCA (n = 4–6) and CSMCs (n = 15–17) with bFGF (50 ng/mL and 25 ng/mL, respectively) treatment compared to control. Addition of Apo (1.0 mM) abolished bFGF-induced increases in KCNN4 expression, and alone also significantly decreased basal KCNN4 expression in CSMCs. *P<0.05 vs respective control. #P<0.05 vs respective bFGF.

qRT-PCR was used to determine if NOX is required for bFGF-induced increases in KCNN4 mRNA expression. Cultured CSMCs were treated with bFGF, bFGF+Apo, or Apo for 24 hours. Freshly isolated RCA sections were mechanically denuded to remove the endothelium, placed in RPMI, treated with bFGF, bFGF+Apo, or Apo, and incubated at 37°C for 24 hours. In RCA sections, treatment with bFGF significantly increased KCNN4 expression, which was inhibited by treatment with Apo (Figure 2). In cultured CSMCs, treatment with bFGF significantly increased KCNN4 expression while treatment with Apo inhibited both bFGF increased and basal KCNN4 expression (Figure 2).

### Inhibition of NOX prevented bFGF upregulation of KCNN4 protein expression

**Figure 3:**
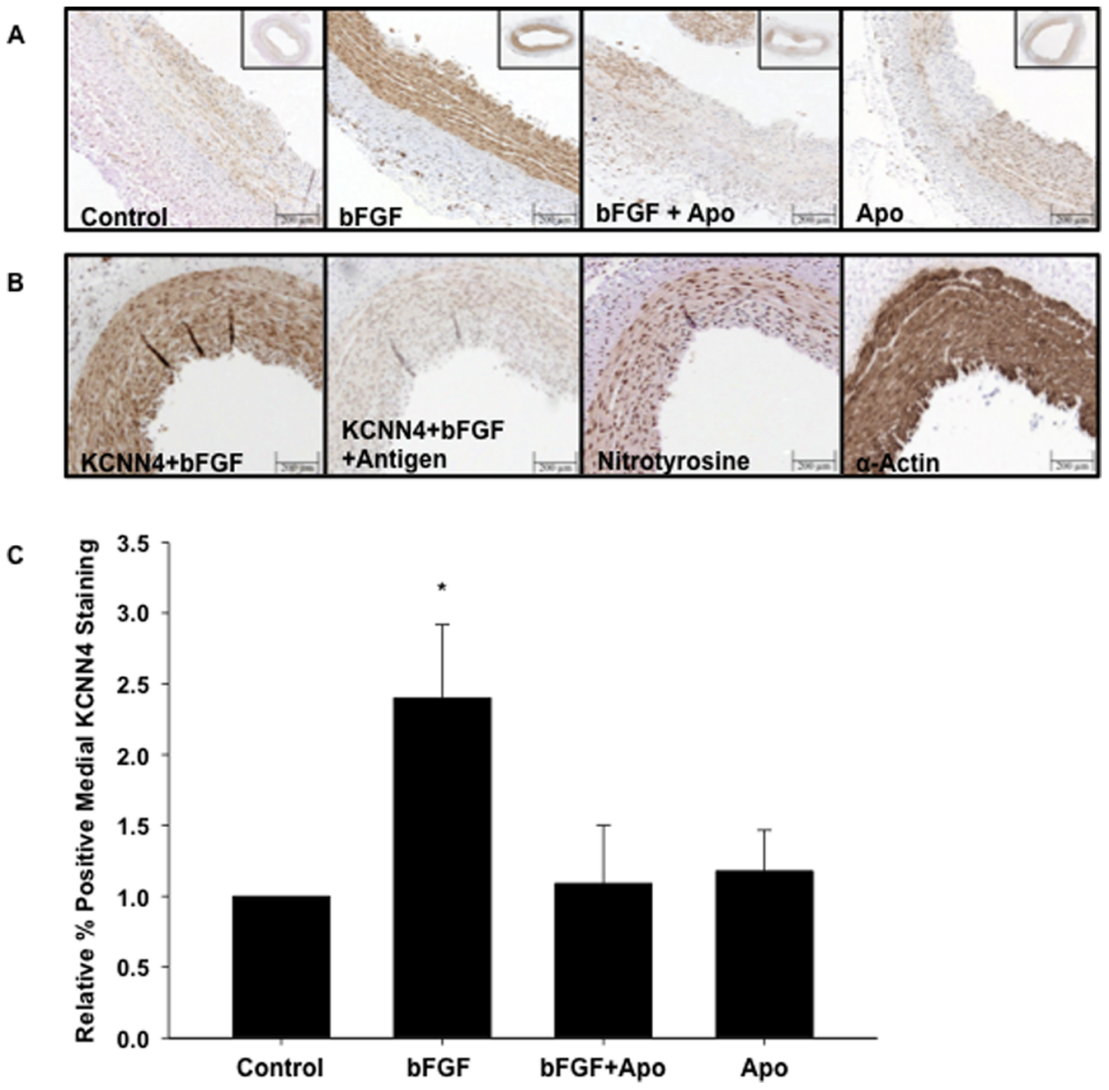
Inhibition of NOX prevented bFGF upregulation of KCNN4 protein expression. Treatment of RCA sections with bFGF (50 ng/mL) caused a significant increase in medial KCNN4 protein expression, while addition of Apo (1.0 mM) inhibited the response to bFGF; (A) representative images and (C) average relative percent positive KCNN4 staining (n = 3). (B) Demonstration of selectivity of anti-KCNN4 staining by antigen pre-absorption and distribution of nitrotyrosine and SM-actin. Positive staining is brown in all images. *P<0.05 vs control.

To determine if NOX-mediated, bFGF-induced increases in KCNN4 mRNA translate to increased protein expression, freshly isolated RCA sections were treated with bFGF, bFGF+Apo, or Apo for 24 hours, formalin fixed, and sectioned. Medial KCNN4 protein expression was increased in response to treatment with bFGF, while addition of Apo abolished the bFGF increase in KCNN4 protein expression (Figure 3A, 3C). Sections were also stained with the smooth muscle marker SMαA to confirm the media was primarily composed of CSMCs, and to confirm that the uneven KCNN4 staining pattern was not artifact. Interestingly, the staining pattern of the oxidative stress marker nitrotyrosine was similar to KCNN4, consistent with increased oxidative stress in regions of the media with elevated KCNN4 protein expression (Figure 3B).

### Inhibition of NOX prevented bFGF upregulation of KCNN4 activity

**Figure 4:**
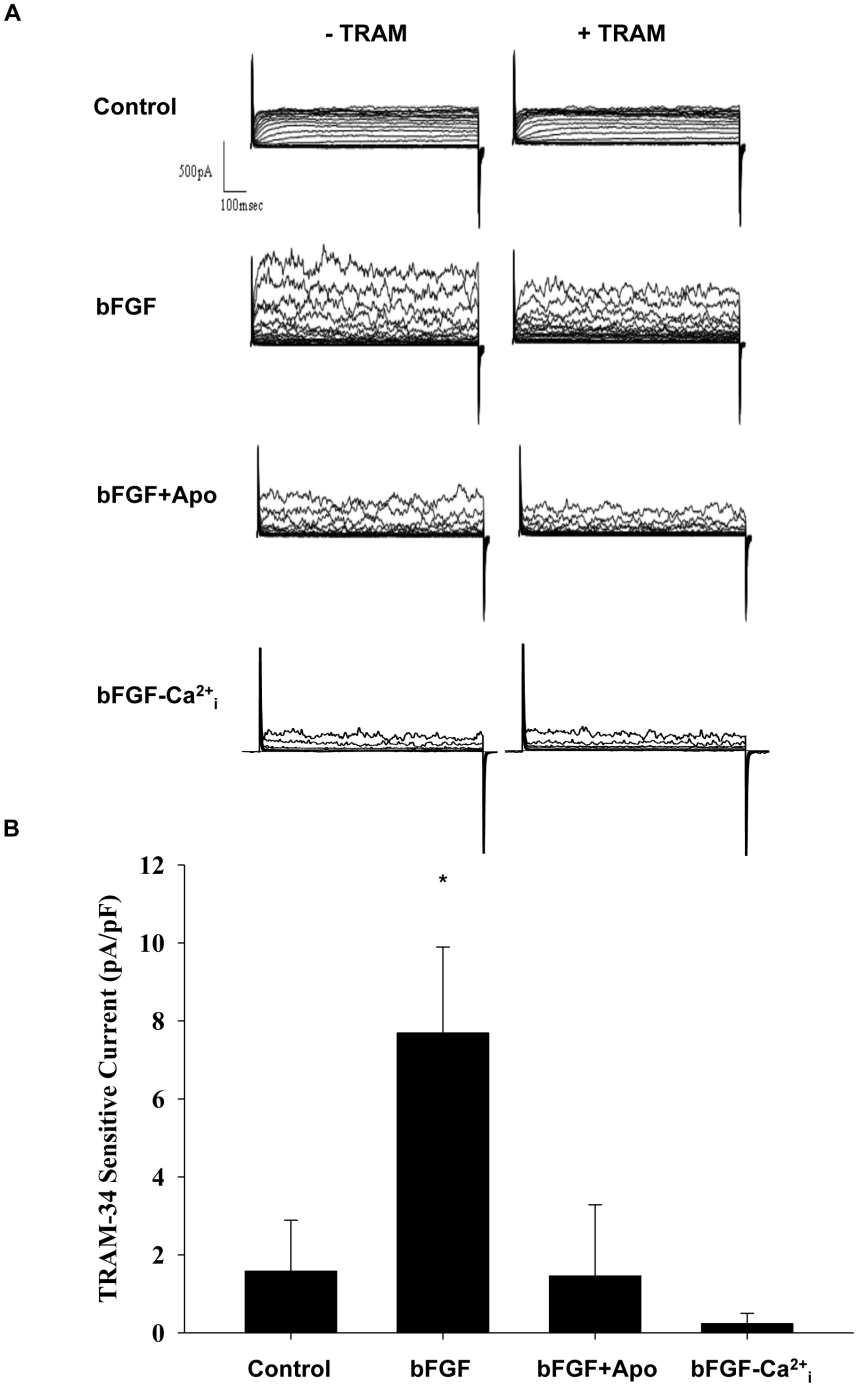
Inhibition of NOX prevented bFGF upregulation of KCNN4 activity. 24-hour treatment with bFGF (25 ng/mL) increased TRAM-34 sensitive K^+^ channel activity, which was inhibited by addition of Apo (1.0 mM; n = 10–17). Reduction of internal calcium prevented bFGF induced channel activity. (A) Representative ensemble step currents (−70 mV to 100 mV) before and after addition of the KCNN4 specific channel blocker TRAM-34 (100 nM), (B) TRAM-34 sensitive currents at 100 mV in CSMCs treated with bFGF or bFGF+Apo, and in internal calcium-free (-Ca^2+^_i_) conditions. *P<0.05 vs control.

Whole-cell voltage clamp was used to determine if NOX-mediated, bFGF-induced upregulation of KCNN4 results in increased KCNN4 channel activity. To isolate current through KCNN4, K^+^ current was measured before and after addition of the specific KCNN4 channel blocker TRAM-34. Widely used as an inhibitor of KCNN4, previous patch-clamp studies have shown that TRAM-34 has a 1,000 times greater selectivity for KCNN4 versus other calcium, potassium, and sodium channels [64], and previous specificity screens comparing 30 receptors and transporters have confirmed that it is a highly specific blocker of KCNN4 [17]. To confirm calcium sensitivity, TRAM-34 sensitive K^+^ current was measured following treatment with bFGF using a nominally calcium free pipette solution. Representative ensemble currents from individual cultured CSMCs (Figure 4A) and TRAM-34 sensitive currents (Figure 4B) demonstrate significantly increased KCNN4 current in response to treatment with bFGF. Consistent with KCNN4 mRNA and protein expression, treatment with Apo prevented bFGF-induced increases in KCNN4 channel activity, indicating that NOX-derived upregulation of KCNN4 results in functional KCNN4 channels at the membrane. Absence of TRAM-34 sensitive current in nominal internal calcium conditions confirmed measurement of calcium-sensitive potassium current (Figure 4).

### NOX regulation of KCNN4 involves the AP-1 transcription factor

**Figure 5:**
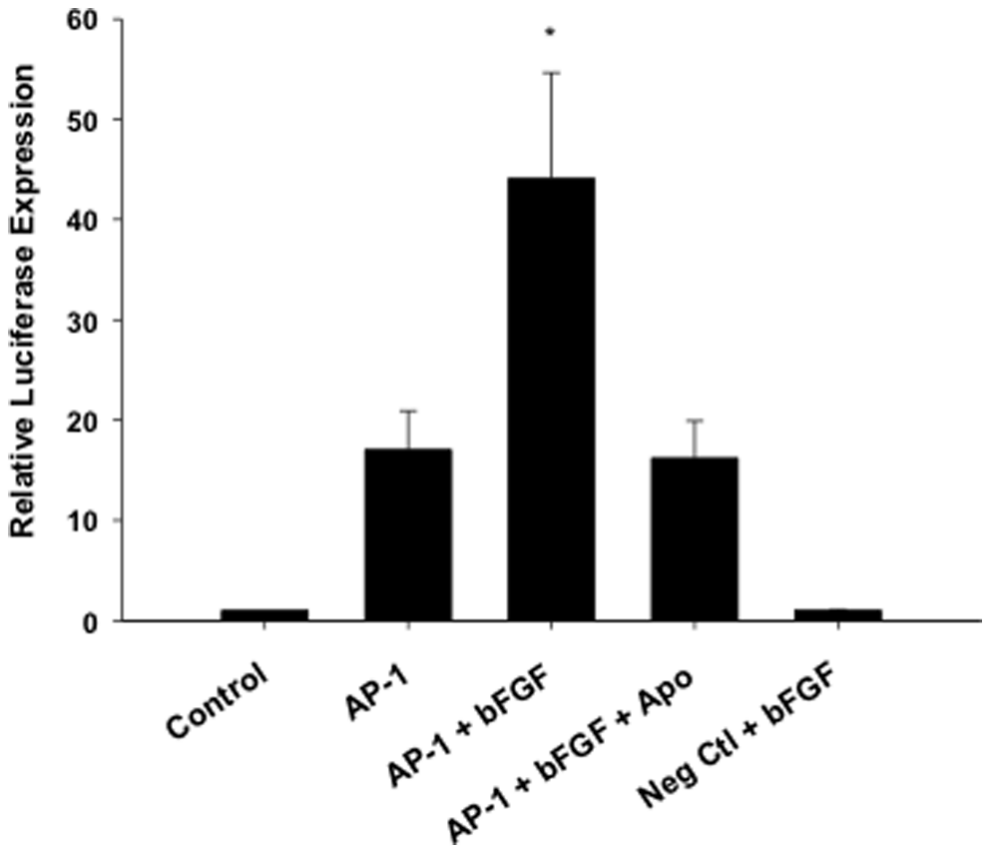
NOX regulation of KCNN4 involves the AP-1 transcription factor. Treatment with bFGF (25 ng/mL) for 24 hours increased AP-1 promoter reporter activity in CSMCs nucleofected with AP-1 luciferase promoter-reporter plasmid. Addition of Apo (1.0 mM) abolished the bFGF increase in AP-1 promoter activity (n = 9–12). Luciferase activity was absent in CSMCs nucleofected with a negative control plasmid (NegCtl). *P<0.05 vs AP-1.

To determine if the AP-1 transcription factor is involved in NOX regulation of KCNN4, cultured CSMCs were nucleofected with pAP-1 Luc Cis-Reporter plasmid, and treated with bFGF, bFGF+Apo, or Apo for 24 hours. Following treatment, firefly luciferase activity was measured and normalized to Renilla luciferase activity. Analysis demonstrated a significant increase in AP-1 promoter reporter activity in response to bFGF, which was inhibited by treatment with Apo (Figure 5).

### Porcine coronary smooth muscle expressed NOX1, NOX2, NOX4, and NOX5 isoforms

**Figure 6:**
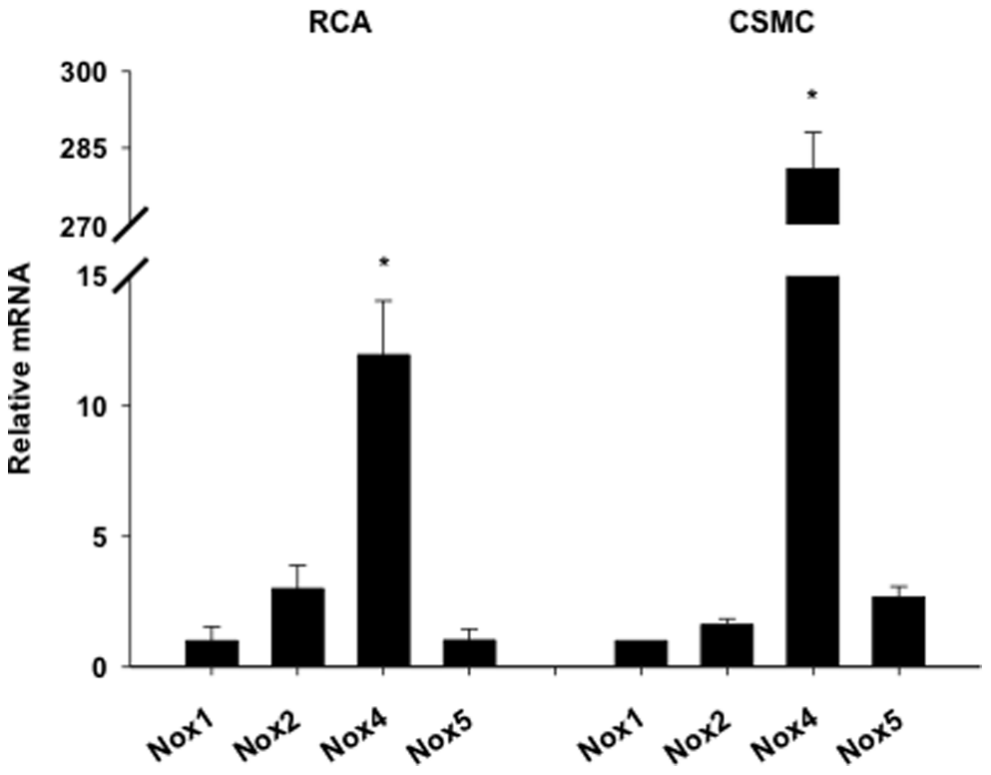
Porcine coronary smooth muscle expresses NOX1, NOX2, NOX4, and NOX5 isoforms. Basal mRNA expression of NOX isoforms 1, 2, 4, and 5 in RCA and CSMCs. NOX4 mRNA expression was significantly higher in both porcine RCA (n = 12–13) and CSMCs (n = 13–15). *P<0.05 vs respective NOX1.

To determine which NOX isoforms are expressed in porcine coronary smooth muscle, freshly isolated RCA sections and cultured CSMCs were harvested as described above. qRT-PCR analysis showed that all four cardiovascular NOX isoforms (NOX1, NOX2, NOX4, and NOX5) were expressed in both RCA and CSMCs, with NOX4 having the highest mRNA expression (Figure 6).

### Knockdown of NOX5 prevented bFGF upregulation of KCNN4 mRNA expression

**Figure 7:**
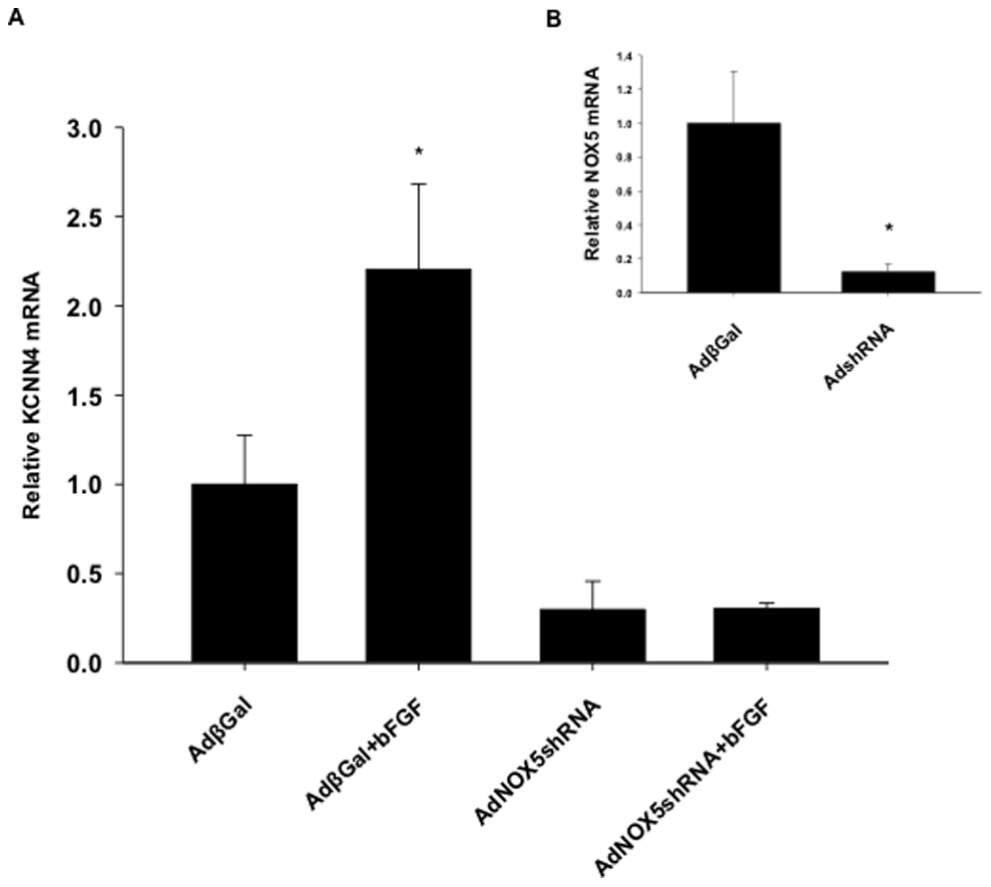
Knockdown of NOX5 prevented bFGF upregulation of KCNN4 mRNA expression. Infection with AdNOX5shRNA significantly reduced NOX5 mRNA expression (B) and prevented KCNN4 mRNA upregulation by bFGF (25 ng/mL) in CSMCs, while the control adenovirus (AdβGal) had no effect (A, n = 4). *P<0.05 vs control.

**Figure 8:**
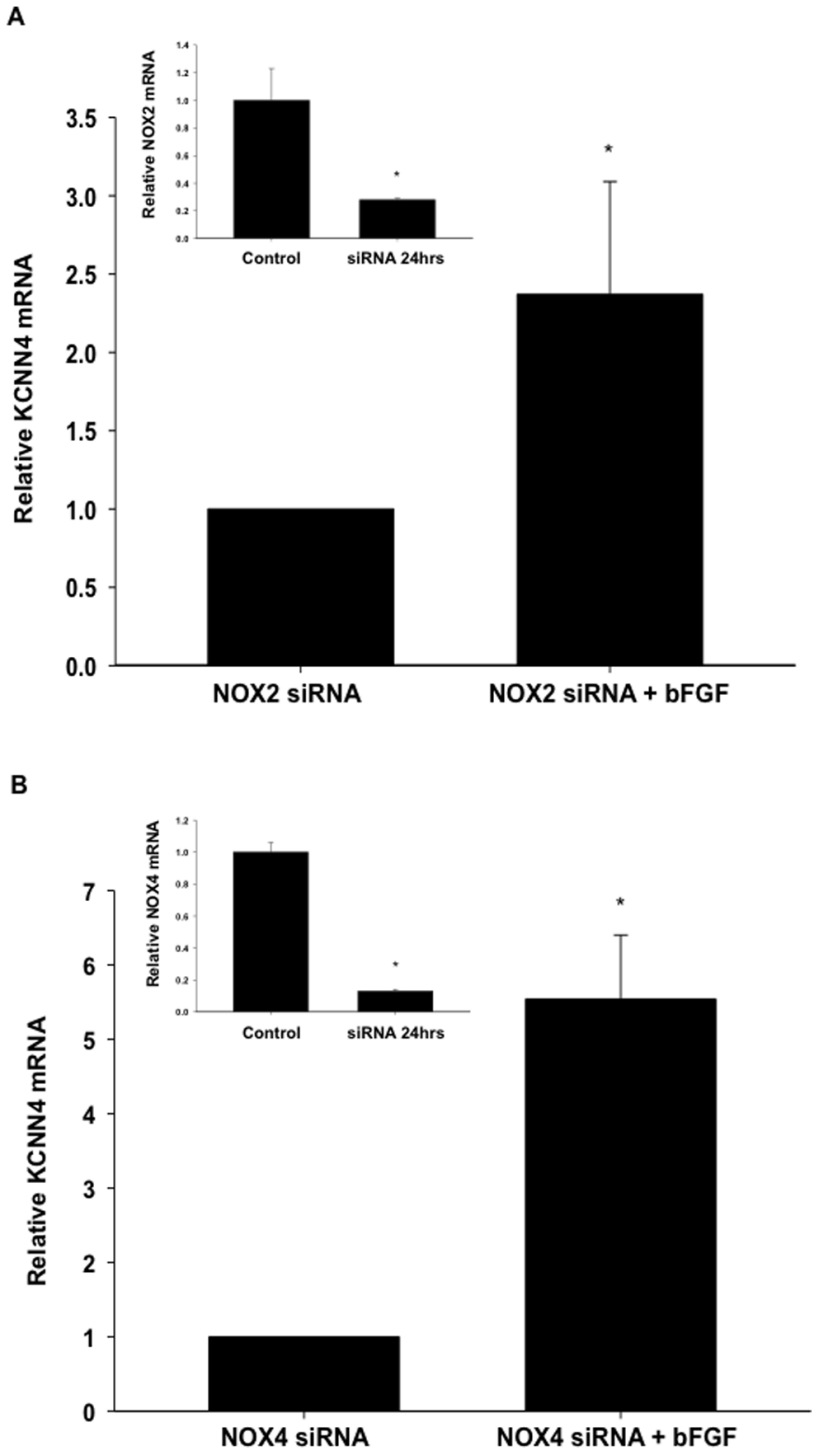
Knockdown of neither NOX2 nor NOX4 prevented bFGF upregulation of KCNN4 mRNA expression. Nucleofection with siNOX2 significantly reduced NOX2 expression but did not prevent upregulation of KCNN4 mRNA expression by bFGF (25 ng/mL) in CSMCs (A, n = 5–9). Similarly, nucleofection with siNOX4 significantly reduced NOX4 expression but did not prevent upregulation of KCNN4 mRNA expression by bFGF (25 ng/mL) in CSMCs (B, n = 5–9). *P<0.05 vs control.

To determine if NOX5 is required for NOX regulation of KCNN4, CSMCs were infected with adenovirus expressing shRNA targeted to knockdown NOX5 (AdNOX5shRNA), treated with bFGF for 24 hours, isolated, and prepped for qRT-PCR. Knockdown of NOX5 (Figure 7B) completely prevented bFGF-induced increases in KCNN4 mRNA expression (Figure 7A). While AdNOX5shRNA reduced NOX5 mRNA expression by ∼90% (Figure 7B), it did not reduce mRNA expression of other NOX isoforms, such as NOX 1, 2 or 4 (0.68±0.27, 0.93±0.47, 0.83±0.07) versus respective controls (1±0.38, 1±0.4, 1±0.1). Furthermore, knockdown of neither NOX2 (Figure 8A) nor NOX4 (Figure 8B) prevented upregulation of KCNN4 mRNA indicating bFGF upregulation of KCNN4 was NOX5 specific.

### Knockdown of NOX5 prevented coronary smooth muscle cell migration

**Figure 9:**
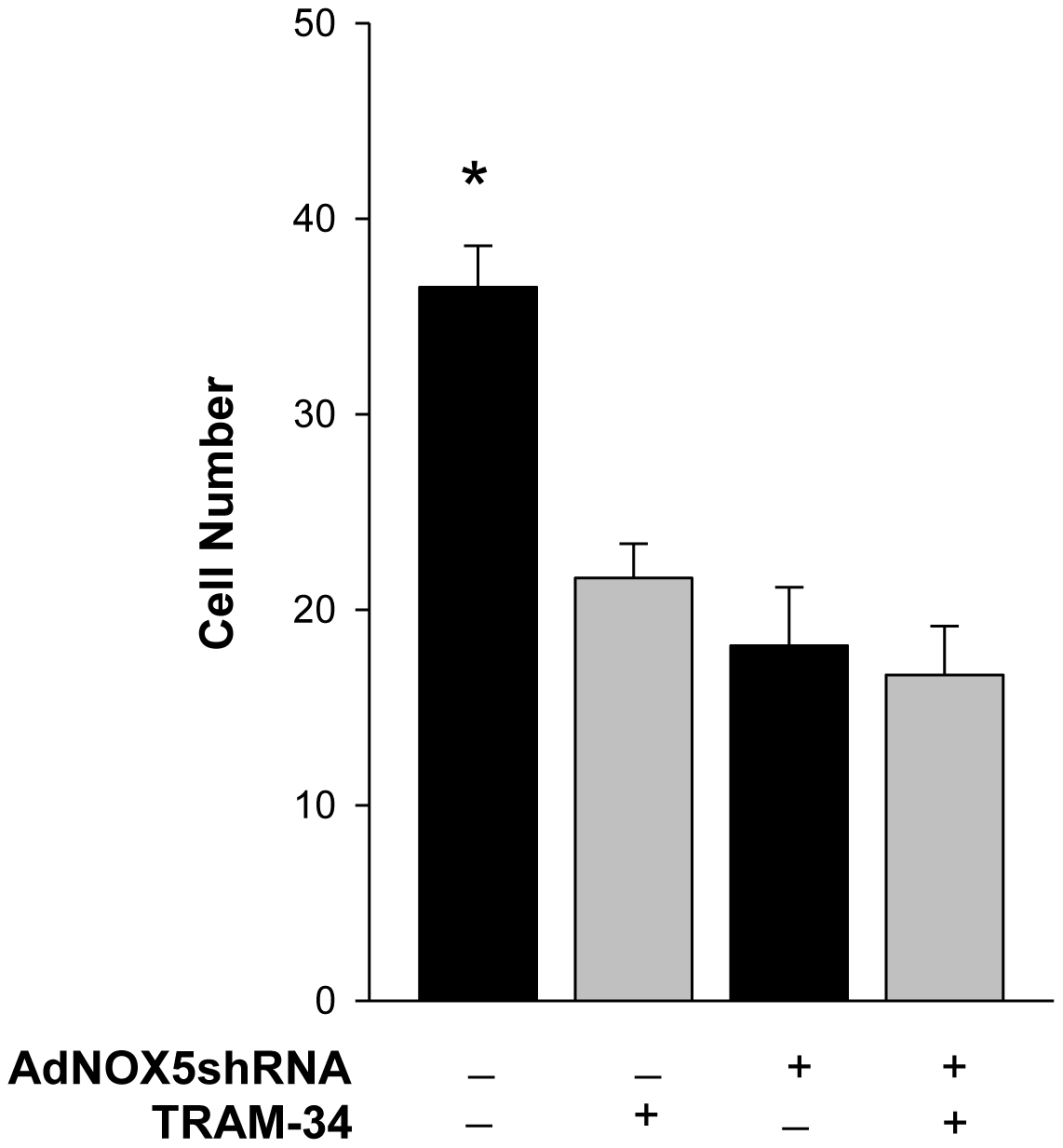
Knockdown of NOX5 prevented coronary smooth muscle cell migration. Transduction with AdNOX5shRNA significantly reduced chemotactic-induced migration in CSMCs compared to control adenovirus. Addition of TRAM-34 (100 nM), inhibited migration ∼50% in control adenovirus cells, but had no effect in AdNOX5shRNA cells. Cell number per 20X field, n = 6–8. *P<0.05 vs other experimental groups.

To determine if NOX5 plays a role in migration, CSMCs were infected with adenovirus expressing shRNA targeted to knockdown NOX5 (AdNOX5shRNA), plated in a chemotaxis chamber, treated with the chemotactic agent PDGF-BB with or without TRAM-34 for 4 hours, stained, and counted (Figure 9). Compared to control adenovirus in the presence of PDGF-BB (36.50±2.11 cells/field, n = 8), knockdown of NOX5 significantly inhibited migration (18.16±2.99 cells/field, n = 6). Lack of further inhibition of migration by TRAM-34 in the NOX5 knockdown group (16.67±2.49 cells/field, n = 6) supports a NOX5-dependent KCNN4 role in CSMC migration.

## Discussion

Oxidative stress induced by ROS plays a key role in atherogenesis and atherosclerotic plaque instability [26], [49], [65], [66]. Increased production of ROS activates reduction-oxidation (redox)-sensitive signaling pathways resulting in endothelial activation, oxidative modification of lipids, proliferation of vascular SMCs, and recruitment of vascular SMCs into atherosclerotic plaques [4], [26], [49]. Studies have shown that the O_2_^.−^ anion is one of the most important ROS in the vasculature [67]. In vascular SMCs, O_2_^.−^ production is increased in response to numerous stimuli including growth factors [34], [38], [68]. Our results demonstrate that treatment with the angiogenic factor, bFGF, significantly increased CSMC O_2_^.−^ production. Previous studies have shown that within the vasculature, NOX is the primary source of ROS including O_2_^.−^ [44], [69], [70]. Consistent with these reports, treatment with the NOX inhibitor Apo abolished both bFGF-stimulated and basal O_2_^.−^ production in CSMCs. Lack of additional inhibition of O_2_^.−^ production by the O_2_^.−^ scavenger Tempol confirms that NOX is the primary enzymatic source of bFGF-stimulated O_2_^.−^ production in CSMCs. It is important to note that an article by Heumueller et al [71] previously questioned the use of Apo as a NOX inhibitor, arguing that the lack of myeloperoxidase (MPO) in vascular SMCs prevented formation of active Apo dimers. Studies have shown, however, that Apo can be activated by alternative peroxidases and MPO secreted by one cell type can be taken up and utilized by an alternative cell type [72].

In recent years, studies have shown that KCNN4 play a key role in the regulation of vascular SMC proliferation and migration associated with atherogenesis, stenosis, and plaque formation [7], [15], [17], [18]. We were able to mimic previous reports of vascular SMC KCNN4 upregulation in response to growth factors [7], , demonstrating increased KCNN4 mRNA expression, protein expression, and channel activity in response to treatment with bFGF. ROS and NOX are known to regulate ion channels, playing a key role in Ca^2+^ signaling and Ca^2+^ release from intracellular stores [40], [74]. Inhibition of the bFGF-induced increase in KCNN4 mRNA, protein, and channel activity by treatment with Apo indicates a critical role for NOX in KCNN4 regulation and provides a novel pathway for NOX to contribute to the progression of atherosclerosis.

Multiple transcription factors including AP-1, NFκB, and c-Jun NH2-terminal kinase (JNK) are redox-sensitive and activated during atherosclerosis [70], [75], [76], [77], [78]. Interestingly, previous studies have shown that growth factor induced upregulation of KCNN4 occurs via transcriptional activation of AP-1 [7], indicating that AP-1 plays a key role in vascular SMC proliferation and phenotypic modulation. Our findings that Apo inhibits bFGF-induced increases in AP-1 promoter activity are consistent with previous reports of AP-1 involvement in oxidative stress [75], [79], and provide evidence of a novel mechanism for NOX regulation of coronary smooth muscle KCNN4. It is important to note that while a previous study by Lapperre et al. (1999) showed upregulation of AP-1 activity following Apo treatment alone [80], our results are similar to a more recent study showing Apo inhibits pro-inflammatory factor-induced AP-1 and NF-B activation [81].

While it is clear that NOX plays a role in cardiovascular disease, including atherosclerosis, the role of the individual NOX isoforms is not as clear. Our results indicate that similar to the human cardiovascular system, porcine coronary smooth muscle expresses NOX1, NOX2, NOX4 and NOX5. Consistent with previous reports, in the basal state NOX4 expression was significantly greater than the other isoforms [43], [82], [83]. To our knowledge this is the first study to show the NOX isoform expression profile of porcine coronary smooth muscle and is of particular interest considering rodents do not express NOX5 [44], [53].

Unlike the other NOX isoforms, NOX5 is composed of four cytosolic EF-hands with Ca^2+^ binding sites resulting in Ca^2+^ dependent regulation without the need of additional cytosolic subunits for activation [43], [52], [53], [84]. Studies have shown that NOX5 activity and expression is regulated by NF-κB, AP-1, and STAT1/STAT3, and is increased by growth factors including PDGF [52], [85], [86]. NOX5 is thought to be an important regulator of numerous vascular pathologies playing a role in cell proliferation, cell growth, and signal transduction [43], [87]. Combined with previous findings that NOX5 expression is elevated in atherosclerotic plaques [51], [53], our novel findings that knockdown of NOX5 prevents bFGF upregulation of KCNN4 mRNA expression provide additional support that NOX5 plays a role in CVD by regulating vascular SMC proliferation and migration.

Although we demonstrated a complete inhibition of KCNN4 upregulation with NOX5 knockdown, we examined the potential role of other isoforms. NOX2 is the most widely distributed isoform and has been shown to play a role in neointimal formation and restenosis [50], [84], [88], however siRNA knockdown of NOX2 did not prevent bFGF upregulation of KCNN4 mRNA expression in our studies. Consistent with previous reports that NOX4 is downregulated during vascular growth and is thought to be primarily responsible for basal cellular O_2_^.−^ production [9], [26], [44], [89], our findings demonstrate that knockdown of NOX4 does not prevent bFGF upregulation of KCNN4 mRNA expression in CSMCs. These results support previous findings that while increasing ROS production through an NADPH oxidase dependent pathway, bFGF does not upregulate the NOX2 or NOX4 isoforms in human or mouse endothelial cells [90]. Inhibition of NOX1, implicated in the pathogenesis of restenosis and atherosclerosis, has been shown to significantly inhibit neointimal formation [26], [43], [48], [91], and unlike NOX2 and NOX4, has been shown to be upregulated by bFGF in rat and mouse aortic SMCs [92]. Unfortunately we were unable to successfully knockdown NOX1 in porcine CSMCs due to the lack of a complete mRNA sequence for porcine NOX1, thus preventing us from completely ruling out a contribution of NOX1 in bFGF upregulation of KCNN4 mRNA expression.

It is important to note, however, that none of the above mentioned studies examined the role of NOX5 in bFGF-induced ROS production. This is critical considering individual cells express multiple NOX isoforms suggesting separate distinct functions based on subcellular localization, cell type, and stimulus. The individualized functions of the NOX isoforms along with our data demonstrating a complete block of KCNN4 upregulation by NOX5 knockdown and high specificity of NOX5 mRNA knockdown by AdNOX5shRNA do, however, strongly suggest little to no role for other isoforms including NOX1 in bFGF-induced upregulation of KCNN4 in porcine CSMCs.

Based on previous studies looking at the mechanisms of mitogen-induced NOX activation, the potential signaling pathways involved in NOX5 dependent upregulation of KCNN4 in response to bFGF are likely complex and may involve positive feedback loops. bFGF is known to act through a number of signaling pathways including the ras/raf/MEK/ERK/MAPK cascade, the PIP2/IP3 cascade, and a pathway involving PI3K, PKC, and Rac [73], [93], [94]. Although known to result in increased NADPH oxidase activity, the PI3K pathway likely doesn’t play a considerable role in our particular study considering NOX5 activation is calcium sensitive and does not involve cytosolic subunits such as Rac [53].

The PIP2 pathway is of interest though as it has been shown to result in increased cytosolic Ca^2+^ [95], which is known to activate NOX5 [43], [51] as well as KCNN4. Interestingly, studies have shown that IP3 sensitivity is increased by ROS [96], [97] providing a mechanism by which increased NOX5 dependent ROS production may stimulate continued activation of NOX5 and continued release of Ca^2+^ through a positive feedback loop. Another pathway of interest that may be working in conjunction with the PIP2/IP3 cascade is the ras-MAPK signaling pathway. Shown to be activated by growth factors and ROS, and thus potentially NOX5, the MAPK pathway involves activation of the c-jun and c-fos transcription factors inducing AP-1 activity known to be involved in KCNN4 upregulation [23], [98], [99]. KCNN4 activation results in hyperpolarization causing calcium influx [14], which may in turn activate NOX5 as well as further activating KCNN4 through a positive feedback loop.

Based on our previous studies showing upregulation of KCNN4 mediates coronary smooth muscle phenotypic modulation and contributes to stenosis, we conducted chemotaxis assays to determine if NOX5 plays a role in migration. Consistent with previous studies indicating ROS play a role in atherosclerosis and restenosis [26], [27], our findings demonstrate that knockdown of NOX5 inhibits mitogen-induced CSMC migration.

The novel findings of our study, combined with results from previous studies showing KCNN4 plays a key role in regulating SMC phenotypic modulation [7] and post-angioplasty restenosis [18], strongly support a role for NOX5 regulation of KCNN4 in the development of atherosclerosis and restenosis. Moving forward more studies will be necessary to determine the roles of the different signaling pathways discussed, as well as the potential therapeutic benefits of NOX5 inhibition in preventing growth factor-induced vascular SMC proliferation and migration associated with cardiovascular disease.
